# Wide-pulse, high-frequency, low-intensity neuromuscular electrical stimulation has potential for targeted strengthening of an intrinsic foot muscle: a feasibility study

**DOI:** 10.1186/s13047-018-0258-1

**Published:** 2018-05-03

**Authors:** Darren C. James, Matthew C. Solan, Katya N. Mileva

**Affiliations:** 1 Sport & Exercise Science Research Centre, School of Applied Sciences, London South Bank University, 103 Borough Road, London, SE1 0AA UK; 2 Department of Trauma and Orthopaedic Surgery, Royal Surrey County Hospital, Guildford, Surrey, GU2 5XX UK

**Keywords:** Neuromuscular electrical stimulation, Stimulation frequency, Abductor hallucis, Muscle fatigue, M-wave, Motor evoked potential

## Abstract

**Background:**

Strengthening the intrinsic foot muscles is a poorly understood and largely overlooked area. In this study, we explore the feasibility of strengthening *m*. abductor hallucis (AH) with a specific paradigm of neuromuscular electrical stimulation; one which is low-intensity in nature and designed to interleave physiologically-relevant low frequency stimulation with high-frequencies to enhance effective current delivery to spinal motoneurones, and enable a proportion of force produced by the target muscle to be generated from a central origin. We use standard neurophysiological measurements to evaluate the acute (~ 30 min) peripheral and central adaptations in healthy individuals.

**Methods:**

The AH in the dominant foot of nine healthy participants was stimulated with 24 × 15 s trains of square wave (1 ms), constant current (150% of motor threshold), alternating (20 Hz–100 Hz) neuromuscular electrical stimulation interspersed with 45 s rest. Prior to the intervention, peripheral variables were evoked from the AH compound muscle action potential (M_wave_) and corresponding twitch force in response to supramaximal (130%) medial plantar nerve stimulation. Central variables were evoked from the motor evoked potential (MEP) in response to suprathreshold (150%) transcranial magnetic stimulation of the motor cortex corresponding to the AH pathway. Follow-up testing occurred immediately, and 30 min after the intervention. In addition, the force-time-integrals (FTI) from the 1st and 24th WPHF trains were analysed as an index of muscle fatigue. All variables except FTI (T-test) were entered for statistical analysis using a single factor repeated measures ANOVA with alpha set at 0.05.

**Results:**

FTI was significantly lower at the end of the electrical intervention compared to that evoked by the first train (*p* < 0.01). Only significant peripheral nervous system adaptations were observed, consistent with the onset of low-frequency fatigue in the muscle. In most of these variables, the effects persisted for 30 min after the intervention.

**Conclusions:**

An acute session of wide-pulse, high-frequency, low-intensity electrical stimulation delivered directly to abductor hallucis in healthy feet induces muscle fatigue via adaptations at the peripheral level of the neuromuscular system. Our findings would appear to represent the first step in muscle adaptation to training; therefore, there is potential for using WPHF for intrinsic foot muscle strengthening.

**Electronic supplementary material:**

The online version of this article (10.1186/s13047-018-0258-1) contains supplementary material, which is available to authorized users.

## Background

The plantar intrinsic foot muscles originate and insert below the ankle joint complex, and collectively function to provide a local stability to the foot [1, 2]. Alterations in structure, and compromised strength and tonic control in these muscles underlie a variety of common foot pathologies [3–7]. Correspondingly, like any other muscle subjected to repetitive strain, the intrinsic foot muscles require targeted muscle strengthening. However, this remains a poorly understood and largely overlooked area.

Certain voluntary exercises that target the intrinsic foot muscles, in particular *m*. abductor hallucis (AH), have been shown to produce structural and functional adaptations [8, 9]. For example, the ‘short-foot’ exercise, which aims to reduce the length of the (planted) foot by actively raising the medial longitudinal arch, significantly increases arch height index and dynamic balance control following a 4-week training program [8]. Similarly, the ‘toes-spread-out’ exercise has been shown to significantly increase the cross-sectional area of AH in Hallux Valgus sufferers after 8-weeks of training [9]. Despite the merit of these exercises, approximately 20% of asymptomatic individuals are unable to voluntary activate AH [10] - the strongest plantar intrinsic foot muscle [11], and this is manifested further in pathology (eg, Hallux Valgus) [5]. In light of the functional importance of this muscle [1–3, 6, 12], and for its superficial location along the medial-plantar aspect of the foot, targeted neuromuscular electrical stimulation (NMES) represents an alternative solution for strengthening.

Similar to voluntary exercise NMES can increase neural activation and strengthen human skeletal muscle [13]. The effect of NMES may be further enhanced by utilisation of a wide-pulse (1 ms), high-frequency (100 Hz), low-intensity (< 10%MVC) electrical paradigm (WPHF), which preferentially activates large-diameter Ia muscle afferents, enhances the effective current delivered to spinal motoneurons, and minimises antidromic transmission in motor axons, respectively [14–24]. If delivered to the nerve, WPHF enhances spinal neural drive to a greater extent than direct muscle stimulation [15, 18, 20], and has also been shown to facilitate the corticospinal pathways of both upper [22, 23] and lower extremity [21, 22] muscles. However, the relative discomfort associated with this approach, even at low sub-maximal thresholds, is a disadvantage [15]. WPHF muscle stimulation on the other hand, will still enhance force production from the target muscle via the synaptic recruitment of spinal motoneurons [16]; but will also generate larger contractions than nerve stimulation [20], inducing muscle fatigue more readily with prolonged exposure (~ 30 min) [24, 25]. This is the desirable outcome in order for skeletal muscle adaptations to take place [13]; yet high-frequency electrical stimulation is non-physiological [26] and will promote premature fatigue if delivered in isolation [27]. Therefore, to ensure that WPHF fatigue is physiologically-relevant, alternating low-frequencies (20 Hz–100 Hz-20 Hz) can be incorporated into the electrical paradigm. Indeed, this approach has been shown to offset the precipitous decline in muscle force [27] as well as eliciting more force, per stimulus train, than constant high-frequency stimulation alone [16].

We have previously described alterations in inter-segmental foot kinematics following an acute intervention (~30mins) of direct muscle (alternating) WPHF to AH [28]. In the present study, we use a similar protocol to investigate the central and peripheral neural drive adaptations in AH to evaluate the feasibility of using WPHF as a modality for intrinisic foot muscle strengthening; and potentially as an adjunct treatment for rehabilitation of patients with foot pathology. It was hypothesised that an acute intervention of WPHF would significantly reduce the force output generated by AH and modulate characteristics of the compound muscle action potential (M_wave_) and corresponding twitch force indicative of peripheral fatigue and thus, a training response. We additionally hypothesised no central facilitation of the AH corticospinal pathway, in contrast to previous WPHF (nerve) studies [21–23], because: 1) it would be unexpected in a fatigued state [29]; and 2) the method of delivery does not concur with immediate central adaptation [15].

## Methods

### Participants

Nine healthy male volunteers (mean ± SD: 27.4 ± 8.5 years, 81.7 ± 7.1 kg, 1.80 ± 0.1 m) were informed of the testing procedures and provided written informed consent to participate in the study, which was conducted in accordance with the Declaration of Helsinki. Prior approval had been received from the local University Research Ethics Committee (UREC 1350) and participants reported good health and free from any recent orthopaedic trauma, underlying pathology or neurological problems.

### Study design

Each participant attended a familiarisation session prior to main testing to accustom themselves to the experimental procedures, and for identification of: 1) the motor point area of AH in the dominant foot; 2) stimulation threshold for WPHF delivery during the intervention; and 3) the optimal locations and intensities for peripheral nerve and transcranial magnetic stimulation.

The main testing session was conducted on a separate day and consisted of baseline (PRE) measures of the: (i) compound muscle action potential (M_wave_) evoked through peripheral nerve stimulation of the medial plantar nerve and indicative of peripheral muscle excitability; (ii) time-amplitude parameters of the twitch force evoked by peripheral nerve stimulation and indicative of muscle contractility; and (iii) motor evoked potential (MEP) in response to transcranial magnetic stimulation of the motor cortex area in the contralateral brain hemisphere associated with the dominant foot musculature and indicative of the corticospinal excitability to AH. 24 trains of alternating WPHF was then delivered to AH in the dominant foot of participants, and assessments were re-tested immediately (POST) and at 30 min following (RET) cessation of the intervention period. In all, the main testing session lasted approximately 2-h.

### WPHF intervention

**Fig. 1:**
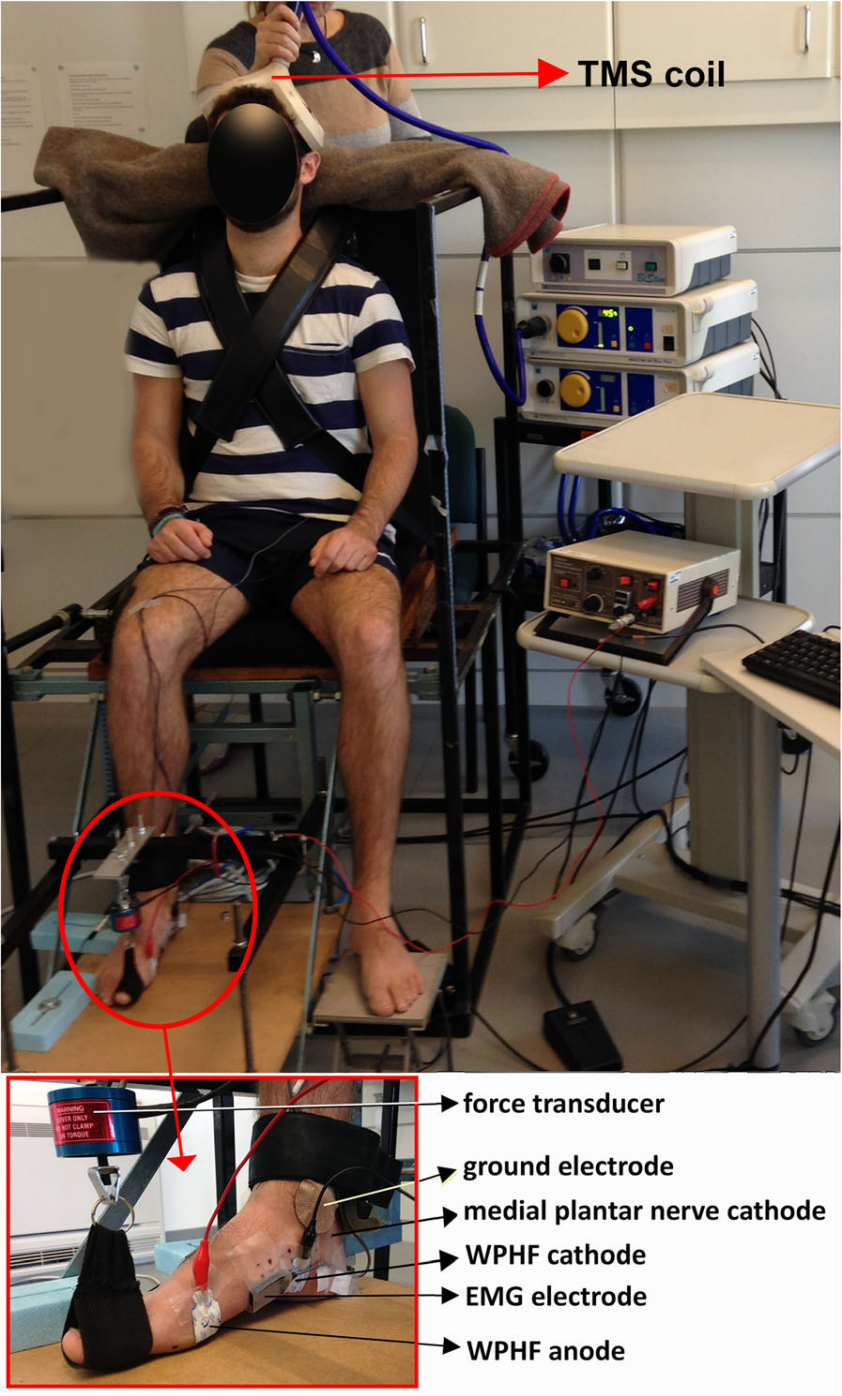
Experimental set-up (demonstrating TMS testing). The insert illustrates the Hallux of the dominant foot suspended from the uni-axial force transducer that was set up to record responses evoked through medial plantar nerve stimulation and delivery of WPHF to the abductor hallucis muscle (the anode for medial plantar nerve stimulation is located on the lateral malleolus)

AH motor point and threshold were identified in the dominant foot during familiarisation for optimal activation and stimulation intensity, respectively. A 7x5cm matrix was drawn with indelible pen over the muscle belly with respect to the navicular tuberosity (Fig. 1) as previously described [28]. Single 500 μs square-wave pulses of 10 mA were delivered systematically to each point of the matrix using a custom built hand-held pen electrode (cathode) from an isolated high-voltage constant current stimulator (DS7A, Digtimer Ltd., Hertfordshire, UK). The motor point area was allocated around the matrix points with largest twitch response measured by the force transducer (Fig. 1). Then, a 2cm^2^ fixed cathode (Ag/AgCl, Cardicare, Cranlea & Co., UK) was attached over this area for the WPHF intervention with the corresponding anode (2cm^2^, Cardicare) positioned over the medial aspect of the distal end of the first metatarsal (Fig. 1). In most cases the motor point area was located 0-1 cm posterior and 3-4 cm distal to the navicular tuberosity. The motor threshold (mA) for AH activation via direct muscle stimulation was then determined by delivering a train of 5x1ms square-wave pulses at 100 Hz. Current intensity started at 1 mA and was increased in 0.5 mA increments until a clear and visible twitch was registered by the transducer, indicative of AH motor threshold. The WPHF stimulation intensity for the intervention was then set at 150% motor threshold [28].

**Fig. 2:**
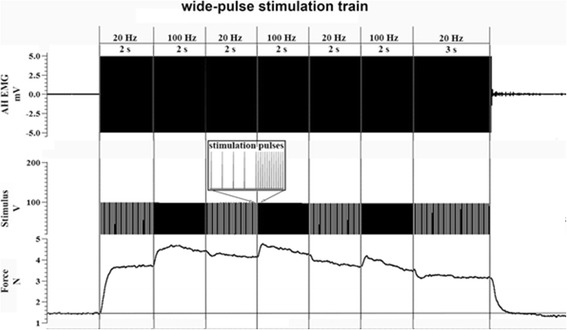
A profile of the 15 s WPHF electrical paradigm delivered at the start of the intervention, and accompanied with corresponding EMG (mainly artefact) and a typical evoked force response (the area represents the force-time-integral). The abductor hallucis of the dominant foot was stimulated with 24 of these trains, each consisting of 2 s alternating periods of 20 Hz and 100 Hz pulses (the last 20 Hz period is 3 s) of 1 ms duration

The location of the 7 × 5 matrix was reproduced at the start of the main testing session and the cathode and anode were re-attached. 24 trains, lasting 15 s each, were then delivered to AH; the trains consisted of square-wave (1 ms[wide-pulse]) pulses delivered in periods of alternating frequencies (20 Hz–100 Hz[high-frequency]-20 Hz; Fig. 2) and interspersed with 45 s rest. The WPHF series were generated by the constant current stimulator, driven by a custom written sequencer using Spike2 data acquisition software (v7.09, Cambridge Electronic Design Ltd., UK) and recorded through an A/D convertor (1401power, Cambridge Electronic Design Ltd., UK).

### Data collection and experimental procedures

One surface EMG electrode in a bi-polar configuration (1 mm width, 10 mm pole spacing; CMRR> 80 dB; model DE2.1, DelSys Inc., USA) was located over the distal aspect of AH, with a reference electrode (Dermatrode, Delsys Inc., USA) attached to the medial malleolus (Fig. 1). The EMG signal was amplified (x1k in 6 and × 0.1 k in 3 participants) and band-pass filtered at the source (20-450 Hz; Bagnoli-8, DelSys Inc., Boston, MA).

A uni-axial force transducer (range: 250 N, RDP Electronics Ltd., UK) was attached to the seating apparatus and suspended the Hallux above the foot supporting surface (Fig. 1). This arrangement permitted unobstructed AH contractions and measurements of the twitch force during the peripheral nerve stimulation testing protocols, and the force exerted by the muscle in response to each WPHF train during the intervention. Twitch responses to TMS were recorded, but not included in the analysis due to the activation of other intrinsic and/or extrinsic foot muscles by this technique.

The EMG and force signals were digitised synchronously via the analogue-to-digital converter, using Spike2 software, with a resolution of 16 bits and a sampling frequency of 2 kHz and 500 Hz, respectively.

#### Peripheral nerve stimulation

**Fig. 3:**
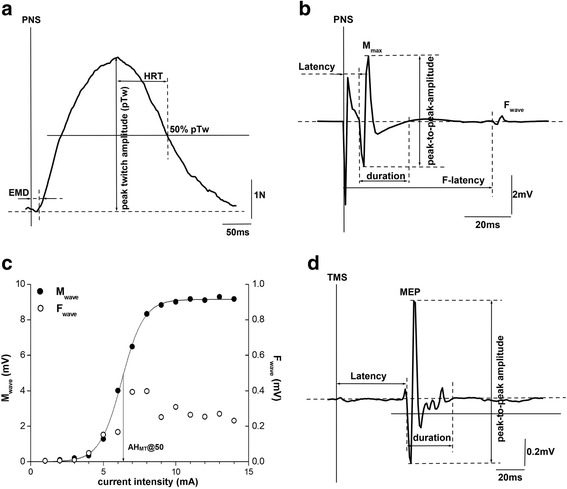
Example of the recorded twitch force (**a**) and M_wave_ (**b**) in response to supramaximal medial plantar nerve stimulation (M_max130_) with designated characteristic time-amplitude parameters. **c** Example of the M_wave_ recruitment curve from increasing (mA) medial plantar nerve stimulation and calculation of AH_MT_@50 (this panel also illustrates the F_wave_ amplitudes at each stimulation intensity). **d** Example of MEP_150_ with designated characteristic time-amplitude parameters. Note: peak-to-peak amplitudes were not entered for statistical analysis; only the respective areas (duration) from the rectified M_max130_ and MEP_150_ profiles

The location of medial plantar nerve stimulation was optimised during the familiarisation visit and corresponded to largest M_wave_ in AH in response to a 500 μs square-wave pulse of 10 mA current (Fig. 1). In main tesing, the amplitude of the maximal M_wave_ (M_max_) was identified during PRE by constructing a recruitment curve in response to three stimulations starting at 1 mA and increasing with 1 mA steps up to saturation of the peak-to-peak M_wave_ amplitude (Fig. 3c). The pulses were delivered using the aforementioned hand-held cathode from the constant current stimulator with the anode (Dermatrode, Delsys Inc., USA) positioned over the lateral malleolus. To assess the intervention-induced changes in peripheral muscle excitability, 3 supramaximal stimulations at an intensity equivalent to 130% of M_max_ (M_max130_; mA) were delivered at PRE, POST and RET.

#### Transcranial magnetic stimulation

Single transcranial magnetic pulses of 100 μs duration and up to 2 T intensity (100% output; Magstim 200, Magstim Co Ltd., UK) were manually triggered and delivered to the motor cortex in the contrlateral brain hemisphere through a hand-held 110° double cone coil (9 cm diameter each, type P/N9902–00, Magstim Co. Ltd) (Fig. 1). The optimal coil position was ascertained for each participant during the familiarisation session using previously reported standards [30]. Thereafter, the motor threshold for activating the motor cortex area relating to AH was established by progressively increasing the pulse intensity until a MEP of minimum 0.2 mV peak-to-peak amplitude in at least 50% of 10 stimulations was established [30]. In main testing, five suprathreshold pulses at 150% motor threshold (MEP_150_; mV) were delivered at random time intervals (at least 10s) to the resting AH during PRE, POST and RET testing periods.

### Data analysis

Data analysis was performed using custom-written scripts developed in Spike2 acquisition software. Prior to extraction of peripheral and central parameters, the DC bias was removed from the EMG signal (time constant of 0.1 s), and the high-frequency noise interferences removed from the force signal (time constant of 0.01 s).

#### Force response to WPHF

The force-time-integral (N.s) from the force signal (Fig. 2) corresponding to the 1st and 24th trains of the WPHF intervention were compared as an index of muscle fatigue.

#### Peripheral muscle excitability

Latency (ms), peak-to-peak amplitude (mV; not reported), total area (mV•ms) and area of the terminal phase (mV•ms) of the recorded M_max130_ were extracted for analysis. Latency was measured between the stimulus event and the beginning of the first positive phase of M_max130_ (Fig. 3b). Total M_max130_ area was calculated from the start of the first positive phase of M_max130_ to the end of the terminal phase in the action potential (Fig. 3b). The area of the terminal phase (M_max130_TP) was calculated in order to infer possible intervention-induced changes in action potential propagation along the muscle fibre membrane [31].

AH excitability was also quantified by calculating the intensity (mA) required to elicit an M_wave_ peak-to-peak amplitude equivalent to 50% of the M_max_ (AH_MT_@50) by way of a sigmoidal fit function (Boltzman plot, Origin v6.0, Microcal Software Inc., USA) applied to the M_wave_ recruitment curve at each testing period in the protocol (Fig. 3c).

#### Peripheral contractility

Peak twitch force (pTw; N), electromechanical delay (EMD; ms) and half relaxation time (HRT; ms) of the twitch evoked by M_max130_ were extracted for analysis. EMD was calculated as the latency between the start of M_max130_ and the beginning of the twitch response (Fig. 3a). HRT, as an indicator of fatigue-related changes in muscle relaxation dynamics [32], was measured between pTw and 50% of pTw (with respect to the ascending trace) in the descending force profile.

#### Corticospinal excitability

The three highest peak-to-peak MEP responses were extracted for analysis of latency (ms), peak-to-peak amplitude (mV; not reported) and total area (mV•ms). MEP latency was measured between the stimulus event and the beginning of the first positive MEP phase (Fig. 3d). Total MEP area was calculated between the start of the first positive phase and the end of the evoked potential (Fig. 3d), and then normalised to the respective testing period average measure of M_max130_ (MEP/M_max130_]*100%).

### Statistics

Individual average values (*n* = 9) for each parameter in each time period were confirmed as being normally distributed (Kolmogorov-Smirnov 1-sample test, PASW v18.0, IBM Corp., USA). Thereafter, all statistical comparisons for a WPHF effect were analysed using a single factor (time: PRE vs POST vs RET) repeated measures ANOVA, with inclusion of effect size (ŋ^2^), and corrected for pair-wise comparisons (Holm-Sidak). A paired-samples t-Test was used to identify a time effect in the FTI between the 1st and 24th WPHF train. Significant differences were accepted when *p* ≤ 0.05.

## Results

The average WPHF current intensity was 3.8 ± 1.5 mA. The average current intensity required to elicit M_max130_ was 15.0 ± 3.2 mA, and the average MEP_150_ intensity was 68.6 ± 15.0% of 2 T. Table 1 presents the average values of all parameters at each time period and the statistical results from *post-hoc* pairwise comparisons when a main time effect was evident.

**Table 1:** Mean (±SD, *n* = 9) corticospinal and peripheral excitability, and contractility measures before (PRE), immediately after (POST) and 30 min following cessation (RET) of WPHF. Significant *post-hoc* pairwise comparisons are indicated. ● indicates *p* ≤ 0.05; ●● *p* ≤ 0.01; ●●● *p* ≤ 0.001

	Central		Peripheral						
	Latency (ms)	MEP/M_max130_ (%)	Latency (ms)	M_max130_ (mV•ms)	M_max130_TP (mV•ms)	AH_MR_@50 (mA)	pTw (N)	EMD (ms)	HRT (ms)
PRE	45.9	31.6	7.0	19.5	6.2	7.7	4.3	5.8	82.7
	3.3	16.1	0.8	9.3	3.9	2.0	1.6	1.3	22.8
POST	46.8	29.7	7.5	21.7	7.3	8.5	4.1	6.3	96.2
	3.7	14.7	0.8	10.2	4.4	2.3	1.8	1.6	20.1
RET	46.4	34.4	7.7	22.1	7.9	8.3	4.2	6.5	99.1
	3.6	17.2	1.0	9.6	5.5	1.9	1.5	2.6	21.6
	†●		†●●● ‡●●●	†●	†●	†●● ‡●			†●● ‡●

†: PRE vs. POST; ‡: PRE vs. RET

Significant time effects were observed in M_max130_ latency (F = 29.5, *p* < 0.001; ŋ^2^ = 0.79), total area (F = 3.81, *p* < 0.05; ŋ^2^ = 0.32), and AH_MT_@50 (F = 15.44, *p* < 0.001; ŋ^2^ = 0.69). Latency and AH_MT_@50 remained significantly elevated in RET. The total area of M_max130_TP showed a tendency for a main effect (M_max130_TP; F = 2.8, *p* = 0.09; ŋ^2^ = 0.26), which in pairwise comparisons revealed a significantly greater area in POST, when compared to PRE (*p* < 0.05). Similar to total area, no retention was observed for this variable (*p* > 0.05).

The evoked muscle twitch force showed a significant time effect for HRT (F = 7.23, *p* < 0.01; ŋ^2^ = 0.48), which also remained significantly prolonged in RET. No time effects were observed for pTw or EMD (*p* > 0.05). A significant time effect was observed for MEP_150_ latency (F = 3.1, *p* < 0.05; ŋ^2^ = 0.35), but not MEP_150_/M_max130_ (*p* > 0.05).

**Fig. 4:**
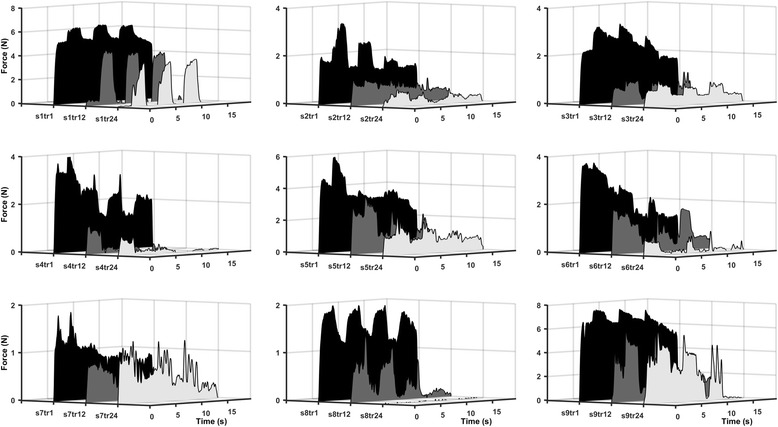
Individual (s1 – s9) WPHF-evoked force-time-integral profiles during the 1st (tr1; black), 12th (tr12; dark grey) and 24th (tr24; light grey) trains of the intervention. These results demonstrate the varied responses of participants to WPHF, although it is clear that the alternating 100 Hz stimulation increases force output during the 1st trial; and in most, this continues for the duration of the intervention. Note the decay in force during 20 Hz stimulation as time progresses (s1, s2, s3, s6, s7, s8); this is indicative of low-frequency fatigue

The WPHF intervention caused significant muscle fatigue. The FTI during the last (24th) train of the intervention was significantly lower compared to that evoked by the first train (11.3 ± 11.8 N•s vs 45.0 ± 29.5 N•s; *p* < 0.01; Fig. 4).

## Discussion

The ability of AH to produce force in response to an acute session of WPHF significantly declined throughout the intervention. The mechanisms underlying this development of fatigue point to peripheral, rather than central processes. Specifically, our findings concur with: (i) a significantly delayed transmission of the action potential along the motoneuron / muscle sarcolemma evidenced by an increased M_max130_ latency and total area of the compound muscle action potential (M_max130_; peak-to-peak amplitude did not change, but the area of M_max130_TP did); (ii) a significantly reduced muscle excitability evidenced by an increased motor threshold (AH_MT_@50); and (iii) a significant impairment in muscle contractility evidenced by a prolonged twitch relaxation (HRT). In most variables this effect persisted for 30mins after the intervention.

The increased MEP_150_ latency was confirmed of peripheral origin after a follow-up evaluation of the F_wave_ latency. The F_wave_ is produced by antidromic activation of motoneurons following distal motor nerve electrical stimulation and provides a means of assessing the excitability of the peripheral nerve between the spinal cord and the target muscle [33]. The latencies recorded at PRE (50.9 ± 0.2 ms), POST (51.0 ± 0.1 ms) and RET (51.1 ± 0.2 ms) allowed us to subsequently calculate peripheral conduction time (PCT: 0.5*((latency of M_max130_ + latency of F_wave_) – 1) and central motor conduction time (CMCT: latency of MEP_150_ - PCT) [34]. There was no difference between testing periods in CMCT (*p* > 0.05); whereas a strong time effect was found in PCT (F = 16.84, *p* < 0.001; ŋ^2^ = 0.68), demonstrating that the WPHF-induced lengthening in MEP_150_ latency was due to a disturbance in the transmission of the motor-evoked potential distal to the site of medial plantar nerve stimulation.

The absence of a central adaptation is in contrast to previous work, which has demonstrated increased excitability in the corticospinal drive to lower leg and hand muscles following an acute session of WPHF to the respective *nerve* [21–23]. *Nerve* vs *direct-muscle* stimulation might explain this disagreement between studies [15]; though other factors including (but not limited to): the electrical paradigm (continuous vs alternating high frequencies), its duty-cycle, and the susceptibility of our participants to respond to the intervention [24] should also be considered. It is noteworthy however, that the relative discomfort associated with *nerve* stimulation, even at sub-maximal thresholds [15], is a disadvantage, and this may limit the clinical utility of the intervention. Direct *muscle* stimulation on the other hand, at low-intensity - as utilised in the present study, avoids the discomfort associated with activating several muscles with differing motor thresholds and evokes contractions of the target muscle only.

The WPHF intervention involved alternating delivery of low (20 Hz) and high (100 Hz) stimulus frequencies. An electrical paradigm such as this evokes more force than constant high-frequency trains alone [16], since low frequency stimulation modulates force output following sustained periods of high frequency activity [15, 16, 27]. In general, force output increased in response to the alternating 100 Hz trains (see Fig. 4 and Additional file 1), which we believe was due to the asynchronous discharge of spinal motoneurons in response to high-frequency stimulation [14–16, 19, 20]. However, a uniform FTI pattern did not exist between participants (Fig. 4), as previously noted [24]. In some, a ‘top-hat’ pattern was observed in the early trains of the intervention with sustained force output during both 20 Hz and 100 Hz stimulation; whereas others exhibited a precipitous decline in force in response to high-frequency stimulation, which was arrested only by the next 20 Hz stimulation. As the intervention progressed in time though, we observed: (i) a progressive decrease in the FTI for each 15 s train; (ii) the profile of force output became irregular and highly variable for both stimulus frequencies; and (iii) in all participants, the magnitude of force output from the ‘physiologically-relevant’ 20 Hz stimulation decayed until minimal, or no force was produced by AH (Fig. 4). Collectively, these features point to the gradual onset of low-frequency fatigue [35] induced by the WPHF intervention.


Additional file 1:Exemplar response to an early 15 s WPHF train. (MP4 21873 kb)


Low-frequency fatigue slows the conduction of the action potentials along the distal motor nerve and muscle membrane, and reduces the excitability of the muscle [36]; our findings from M_max130_ confirmed this happened. In addition, an acute disruption in the excitation-contraction coupling process occurs through elevated intracellular [Ca^2+^] and a subsequent smaller Ca^2+^ transient to and from the sarcoplasmic reticulum [32]; again our findings from HRT suggest this happened [32]. What is important here is that these processes, and in particular an elevated intracellular [Ca^2+^], can be seen to represent the first step in muscle adaptation to training, since this allows for an increasing rate of protein synthesis to take place within the muscle [37]. With regular WPHF application, one might expect to create a environment for muscle hypertrophy and phenotype adaptations [13]; however, these chronic adaptations have only previously been shown in response to high-intensity, high-frequency neuromuscular electrical stimulation. Therefore, our findings suggest a potential for using WPHF for intrinsic foot muscle strengthening, though further work is needed to understand the long-term effects of WPHF, and to optimise the method of WPHF delivery before it can be used outside of a laboratory setting.

The clinical implications of this work are not trivial. Hallux Valgus (bunion) is a forefoot deformity characterized by a lateral deviation of the Hallux and excessive bone proliferation at the dorsomedial aspect of the 1st metatarsal head. Early in its development, morphological changes occur in AH leading to a progressive weakening and loss of function [38], culminating in a significantly atrophied muscle (*p* < 0.001; all grades) when compared to healthy feet [5]. This contributes to significant functional disability, including foot pain [39], impaired gait pattern, postural instability and an increased likelihood of falling in the elderly [40]. Currently, re-constructive surgery remains the only option to permanently correct the deformed Hallux and alleviate these symptoms. Whilst we would not expect the WPHF intervention to have a comparative efficacy to surgical intervention, we do have optimism that long-term use will evoke structural change in the Hallux, similar to what has been shown with voluntary exercise [9], and potentially be capable of offsetting the insidious nature of the deformity and associated pain. Future work will aim to establish at which developmental stage of Hallux Valgus this intervention is most effective.

Finally, there are study limitations to consider. Firstly, identification of the correct location for medial plantar nerve stimulation is subjective. Generally speaking, we observed during the optimisation proceedures (see Methods: *Peripheral Nerve Stimulation*) that it was possible to elicit a larger AH compound muscle action potential from an innervation zone distal to the medial malleolus, but this resulted in predominant flexion movement of the Hallux as well as in the lesser digits. This reflects simultaneous activation of *m*. flexor digitorum brevis and *m*. flexor hallucis brevis. Instead, we opted for a stimulation site distal and posterior to the malleolus (Fig. 1) that evoked a comparatively lower M_wave_, but which resulted in an abduction/plantar movement of the Hallux akin to what would be expected with isolated AH activation. Our findings therefore reflect a preference for contraction specificity over amplitude; however, it is possible, but unlikely (for reasons given below), that our findings underestimate the full contraction capacity of AH.

Secondly, a uni-axial force transducer was used in the present study. This would seem an obvious limitation considering the mechanical function of the Hallux in response to AH stimulation. However, we attempted to negate the possible underestimation of twitch force, and the force recorded from the WPHF intervention, by suspending the Hallux from the transducer - in a sling - above the foot surface. In doing so, we were able to capture part of the abduction movement simultaneously with the plantar movement of the Hallux. Current work in our laboratory using the same procedures, but with a tri-axial transducer, indicates that there is miminal loss in the twitch force recorded from M_max130_ when using a uni-axial transducer. We believe the reason for this could be due to the radiating effect of supramaximal medial plantar nerve stimulation, which ostensibly evokes a plantar movement of the Hallux due to co-activation of the aforementioned intrinsic foot muscles that are innverated by the same nerve. Thus, we believe we have captured close to the true maximum twitch force of AH in the present experimental procedures, but our FTI results during the intervention may be underestimated.

## Conclusions

In summary, our findings show that an acute session of wide-pulse, high-frequency, low-intensity electrical stimulation delivered directly to abductor hallucis in healthy feet induces muscle fatigue via adaptations at the peripheral level of the neuromuscular system. These adaptations are consistent with an immediate response to training; therefore there is potential to evoke strength gains and muscle hypertrophy with chronic application of WPHF. Finally, there is a clinical utility for our electrical paradigm; its method of delivery and stimulus parameters avoid the discomfort commonly experienced with conventional nerve stimulation procedures. Sufferers of Hallux Valgus are the obvious subset of the population who will most benefit from our intervention, and this will be the focus of future work.

## Abbreviations

AH: Abductor hallucis
AH_MT_@50: Abductor hallucis motor threshold at 50% of maximum compound muscle action potential (M_max_)
EMD: Electromechanical delay
FTI: Force-time-integral
HRT: Half relaxation time
MEP_150_: suprathreshold transcranial magnetic stimulation at 150% of the motor evoked potential threshold
M_max130_: supramaximal nerve stimulation at 130% of maximum compound muscle action potential
NMES: Neuromuscular electrical stimulation
pTw: peak twitch force
WPHF: Wide-pulse, high-frequency neuromuscular electrical stimulation
